# Interplay between Oxidative Stress and Metabolism in Signalling and Disease

**DOI:** 10.1155/2016/3274296

**Published:** 2016-01-05

**Authors:** Andrés Trostchansky, Celia Quijano, Hariom Yadav, Eric E. Kelley, Adriana Maria Cassina

**Affiliations:** ^1^Departamento de Bioquímica and Center for Free Radical and Biomedical Research, Facultad de Medicina, Universidad de la República, 11800 Montevideo, Uruguay; ^2^NIDDK, NIH, Diabetes, Endocrinology and Obesity Branch, Bethesda, MD 20892, USA; ^3^Department of Anesthesiology and Vascular Medicine Institute, School of Medicine, University of Pittsburgh, Pittsburgh, PA 15261, USA

It is well recognized that energy metabolism is linked to the production of reactive species and critical enzymatic processes allied to metabolic pathways can be affected by redox reactions. Both protein and lipid oxidation are involved in the aging process as well as the onset and progression of many age-related diseases. As such, the capacity to identify specific targets and detect subsequent oxidative modifications is crucial for the understanding of the molecular basis of age-related diseases (i.e., diabetes, metabolic syndrome, or atherosclerosis) and for revealing novel treatment strategies.

Formation of mitochondrial reactive oxygen and nitrogen species (ROS and RNS, resp.) has been extensively studied in the literature. Moreover, mitochondrial dysfunction is observed in many pathological conditions in addition to an increase in ROS and RNS production. Thus, the initiation and progression of diseases whose pathogenesis involves mitochondrial dysfunction may be modulated by decreasing mitochondrial oxidant formation. However, mitochondria are not the only source of reactive species in cells; for example, catabolism of biomolecules can be a source of oxidant formation with critical intracellular outcomes. For example during *β*-oxidation of fatty acids, the electron transfer flavoprotein (ETF) produces superoxide (O_2_^∙−^) and hydrogen peroxide (H_2_O_2_) upon reduction with its substrate, medium chain acyl-CoA dehydrogenase (MCAD). Superoxide and H_2_O_2_ can be formed in several metabolic pathways where electron transfer reactions are involved and can subsequently lead to the formation of highly oxidizing species such as peroxynitrite and lipid-derived electrophiles. As such, capacity to detoxify reactive species with a battery of antioxidant enzymes is critical in preventing the reactions of oxidants with cellular components.

In this special issue, we present several examples of the interplay between oxidative stress and metabolism.

The manuscript by M. da Cunha and colleagues discusses mitochondria-to-nucleus retrograde signaling in various organisms as well as the differences in effector pathways, molecules, and outcomes. Almost 99% of mitochondrial proteins are encoded in the nucleus; however, mitochondrial DNA does encode some key proteins. The correct communication between mitochondria and the nucleus is seminal in coordinating mitochondrial protein synthesis during biogenesis whereas potential mitochondrial malfunction can influence in this communication. Mitochondrial role in apoptosis, addressed by J. A. Ronchi et al., describes Ca^2+^-dependent opening of mitochondrial membrane permeability transition pore (PTP) and ROS generation. In their manuscript, the authors propose that PTP opening is a relevant process of mitochondrial Ca^2+^ signaling when a redox imbalance is present. The NADPH/NADP^+^ ratio was also analyzed in terms of mitochondrial ROS formation in hypercholesterolemic mice after supplementation with citrate or by inhibition of the NADPH consuming anabolic cholesterol synthesis pathway. Overall, the authors showed a positive correlation of the atherosclerotic lesion with mitochondrial ROS formation in liver.

The NAD-dependent protein deacetylases sirtuins (SIRT) regulate metabolic enzymes maintaining cellular homeostasis. L. Santos et al. present a thorough revision on how sirtuins are regulated at the expression level and/or by proteasomal degradation depending on the degree of oxidative stress. Finally, the authors discuss how SIRT3 may be regulated by oxidant species generated in the mitochondrial matrix.

Santos J. and colleagues addressed nutrient signaling pathways related to caloric restriction (in particular, how aging and caloric restriction interact by overlapping the activation of insulin-derived pathways). Meanwhile, R. Mastrocola et al. analyzed metabolic disorders and its relation with high fat diet. In their original article S. Kun et al. explore how hydroxyl radical- or metabolic-derived Phe or Tyr derivatives alter the gluconeogenic pathway in nondiabetic septic patients and propose that these species may serve as indicators for insulin-based therapies in these patients.

The aim of this special issue was to increase our knowledge of the role of metabolism and oxidative stress in cell signaling and disease, particularly about the molecular mechanisms participating in these processes as well as their role in human diseases and cell physiology.

